# A systematic review and meta-analysis of neopterin in rheumatic diseases

**DOI:** 10.3389/fimmu.2023.1271383

**Published:** 2023-09-18

**Authors:** Arduino A. Mangoni, Angelo Zinellu

**Affiliations:** ^1^ Discipline of Clinical Pharmacology, College of Medicine and Public Health, Flinders University, Adelaide, SA, Australia; ^2^ Department of Clinical Pharmacology, Flinders Medical Centre, Southern Adelaide Local Health Network, Adelaide, SA, Australia; ^3^ Department of Biomedical Sciences, University of Sassari, Sassari, Italy

**Keywords:** neopterin, rheumatic diseases, inflammation, oxidative stress, biomarkers, metabolism

## Abstract

**Introduction:**

Novel biomarkers of inflammation and oxidative stress might enhance the early recognition, management, and clinical outcomes of patients with rheumatic diseases (RDs). We assessed the available evidence regarding the pathophysiological role of neopterin, the oxidation product of 7,8-dihydroneopterin, a pteridine generated in macrophages activated by interferon-γ, by conducting a systematic review and meta-analysis of studies reporting its concentrations in biological fluids in RD patients and healthy controls.

**Methods:**

We searched electronic databases for relevant articles published between inception and 31 August 2023. The risk of bias and the certainty of evidence were assessed using the Joanna Briggs Institute Critical Appraisal Checklist and the Grades of Recommendation, Assessment, Development and Evaluation Working Group system, respectively.

**Results:**

In 37 studies, when compared to healthy controls, RD patients had significantly higher concentrations of neopterin both in plasma or serum (standard mean difference, SMD=1.31, 95% CI 1.01 to 1.61; p<0.001; moderate certainty of evidence) and in the urine (SMD=1.65, 95% CI 0.86 to 2.43, p<0.001; I^2^ = 94.2%, p<0.001; low certainty of evidence). The results were stable in sensitivity analysis. There were non-significant associations in meta-regression and subgroup analysis between the effect size and age, male to female ratio, year of publication, sample size, RD duration, C-reactive protein, erythrocyte sedimentation rate, specific type of RD, presence of connective tissue disease, analytical method used, or biological matrix investigated (plasma *vs*. serum). By contrast, the effect size was significantly associated with the geographical area in studies assessing serum or plasma and with the type of RD in studies assessing urine.

**Discussion:**

Pending additional studies that also focus on early forms of disease, our systematic review and meta-analysis supports the proposition that neopterin, a biomarker of inflammation and oxidative stress, can be useful for the identification of RDs. (PROSPERO registration number: CRD42023450209).

**Systematic review registration:**

PROSPERO, identifier CRD42023450209

## Introduction

Rheumatic diseases (RDs) is an umbrella term that includes a wide number of chronic, disabling conditions characterized by inflammation and oxidative stress affecting the musculoskeletal system and other organ and tissues. Broadly speaking, RDs can have a predominantly autoimmune (e.g., progressive systemic sclerosis, pSS, rheumatoid arthritis, RA, systemic lupus erythematosus, SLE, Sjogren’s syndrome, SSj, systemic sclerosis, SSc, and idiopathic inflammatory myositis, IIM), mixed-autoimmune-autoinflammatory (e.g., ankylosing spondylitis, AS, axial spondylarthritis, axSpA, psoriatic arthritis, PsA, and Behcet’s disease, BD), or autoinflammatory component (e.g., familial Mediterranean fever, FMF) (1–3). The availability of a wide range of anti-inflammatory and immunomodulatory medications has revolutionised the management of clinically overt RDs over the last 20-30 years, with significant improvements in symptom control and quality of life of affected patients (4–7) (8–10). However, despite these advances, significant challenges remain with the identification of early forms of RD. This issue, in turn, prevents the implementation of strategies for the rapid control of dysregulated immune and inflammatory pathways and, potentially, the achievement of more favourable long-term clinical outcomes (11–16). Therefore, a significant body of research has been conducted to identify novel biomarkers of RDs which could better assist physicians to make an early diagnosis, in addition to clinical assessment and conventional biomarkers of inflammation, e.g., C-reactive protein (CRP) and erythrocyte sedimentation rate (ESR) (17–25).

**Figure 1:**
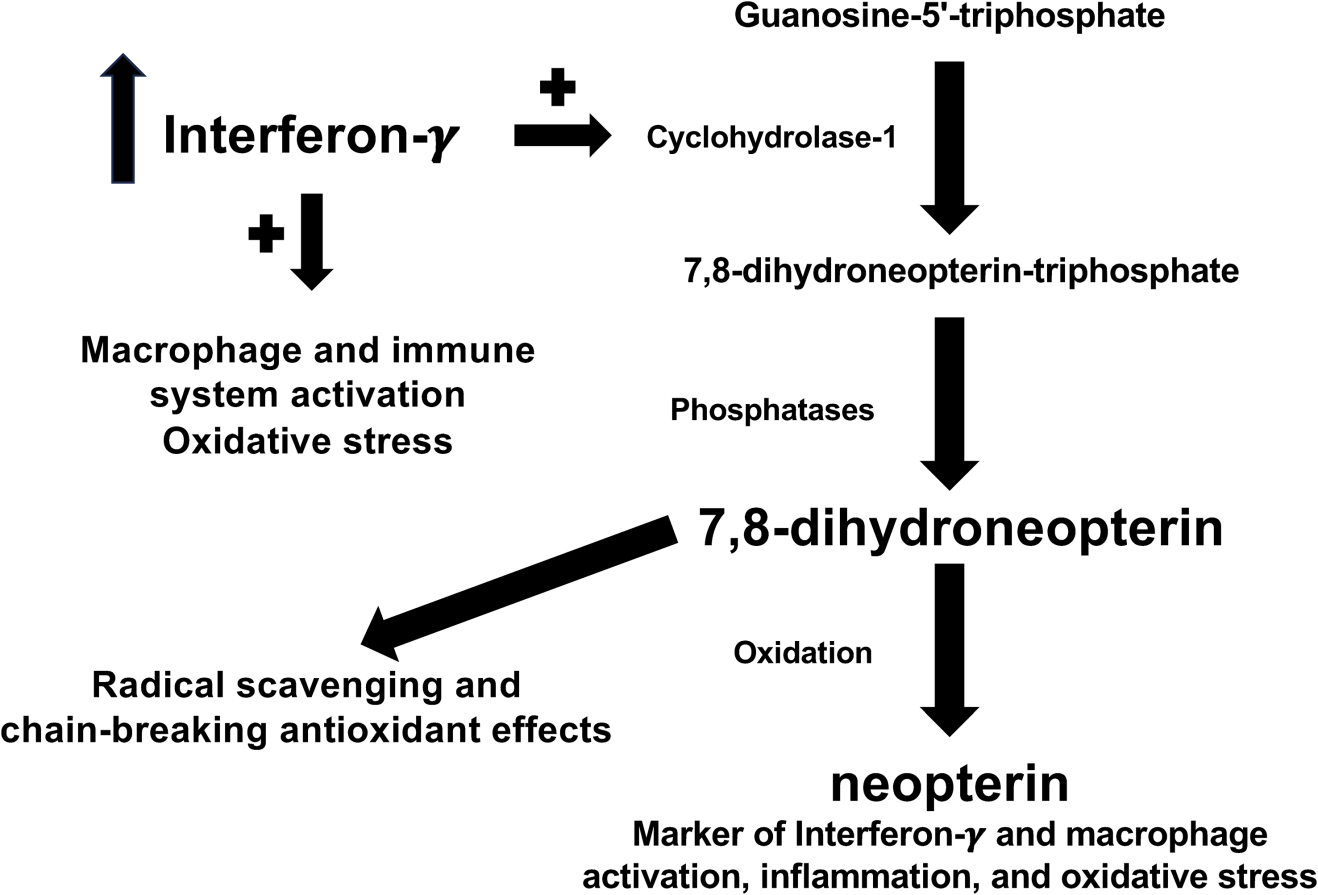
Biochemical pathways involved in the formation of neopterin.

In the ongoing search for novel cellular and biochemical pathways underlying the pathophysiology of RDs, increasing attention has been given to the pleiotropic effects of the cytokine interferon-γ (26). When produced in excess, interferon-γ exerts detrimental effects on the homeostatic control of inflammatory and immune pathways in a range of experimental and clinical studies of RDs (27–30). Therefore, the identification of biomarkers that adequately reflect the activation of interferon-γ might be particularly useful for diagnosis and management. One such biomarker is neopterin, a pteridine analogue generated from the oxidation of 7,8-dihydroneopterin, a potent radical scavenging and chain-breaking antioxidant derived from the interferon-γ-mediated conversion of guanosine-5’-triphosphate (GTP) by GTP cyclohydrolase-1 in activated macrophages (**Figure 1**) (31–34). The potential advantages of measuring neopterin in the clinical evaluation of RDs include, in addition to its role as a marker of macrophage activation, the determination in different biological fluids and its rapid elimination by the kidney, which allows assessing fluctuations in disease activity and early effects of treatment (35–40).

Therefore, we investigated the potential clinical utility of neopterin by conducting a systematic review and meta-analysis of studies investigating the concentrations of this pteridine metabolite in different biological fluids in patients with RD and healthy controls. We also investigated associations between the effect size of the differences in neopterin concentrations and several parameters, including RD duration, type of RD (autoimmune, mixed autoimmune-autoinflammatory, or autoinflammatory disease), CRP, and ESR.

## Materials and methods

### Search strategy, eligibility criteria, and study selection

We systematically searched for relevant publications in the electronic databases PubMed, Web of Science, and Scopus from inception to 31 August 2023 using the following terms and their combination: “rheumatic diseases” OR “rheumatoid arthritis” OR “psoriatic arthritis” OR “ankylosing spondylitis” OR “systemic lupus erythematosus” OR “systemic sclerosis” OR “Sjogren’s syndrome” OR “connective tissue diseases” OR “vasculitis” OR “Behçet’s disease” OR “idiopathic inflammatory myositis” OR “polymyositis” OR “dermatomyositis”AND “neopterin”. Two investigators independently reviewed each abstract and, if relevant, the full-text articles and their references for additional studies. The eligibility criteria included: (i) the assessment of neopterin concentrations in biological fluids (plasma/serum, urine, synovial fluid, saliva, and cerebrospinal fluid, (ii) the comparison between patients with RDs and healthy controls conducted in case-control studies, (iii) the inclusion of patients ≥18 years of age, and (iv) the availability of the full-text of the publication in English language.

The following study and patient variables were independently extracted from selected manuscripts in an *ad hoc* standardized form for further analysis: first author, year of publication, study country, sample size, age, male to female ratio, CRP, ESR, RD duration, sample matrix investigated (serum or plasma), and assay method used to measure neopterin.

We assessed the risk of bias using the Joanna Briggs Institute Critical Appraisal Checklist for analytical studies. Studies addressing ≥75%, ≥50% and <75%, and <50% of checklist items were considered as having a low, moderate, and high risk, respectively (41). We also assessed the certainty of evidence using the Grades of Recommendation, Assessment, Development and Evaluation (GRADE) Working Group system. GRADE assesses the study design (randomized *vs*. observational), the risk of bias (JBI checklist), the presence of unexplained heterogeneity, the indirectness of the evidence, the imprecision of the results (sample size, 95% confidence interval width and threshold crossing), the effect size (small, SMD <0.5, moderate, SMD 0.5-0.8, and large, SMD >0.8) (42), and the probability of publication bias (43). The results were presented according to the guidelines provided in Preferred Reporting Items for Systematic reviews and Meta-Analyses (PRISMA) 2020 statement (**Supplementary Tables 1** and **2**) (44). The review protocol was registered in the International Prospective Register of Systematic Reviews (PROSPERO registration number: CRD42023450209) (45).

### Statistical analysis

We generated forest plots of standardized mean differences (SMDs) and 95% confidence intervals (CIs) to assess differences in neopterin concentrations between RD patients and healthy controls (p<0.05 for statistical significance). If necessary, the mean and standard deviation values were extrapolated from medians and interquartile ranges or medians and ranges (46, 47). The heterogeneity of the SMD across studies was tested by using the Q statistic (p<0.10 for statistical significance). Heterogeneity was considered low when the I^2^ value was ≤25%, moderate when the I^2^ value was >25% and <75%, and high when the I^2^ value was ≥75% (48, 49). A random-effect model based on the inverse-variance method was used in the presence of high heterogeneity. Sensitivity analysis was conducted to investigate the stability of the results by assessing the influence of individual studies on the overall risk estimate (50). Publication bias was assessed using the Begg’s adjusted rank correlation test and the Egger’s regression asymmetry test (p<0.05 for statistical significance) (51, 52). The “trim-and-fill” method was used to further test and eventually correct the occurrence of publication bias (53). Univariate meta-regression and subgroup analyses were conducted to investigate the presence of associations between the effect size (SMD) and the following parameters: year of publication, study continent, sample size, age, male to female ratio, CRP, ESR, disease duration, sample matrix investigated, and analytical method used to measure neopterin. Statistical analyses were performed using Stata 14 (Stata Corp., College Station, TX, USA).

## Results

### Systematic search and characteristics of the included studies

**Figure 2:**
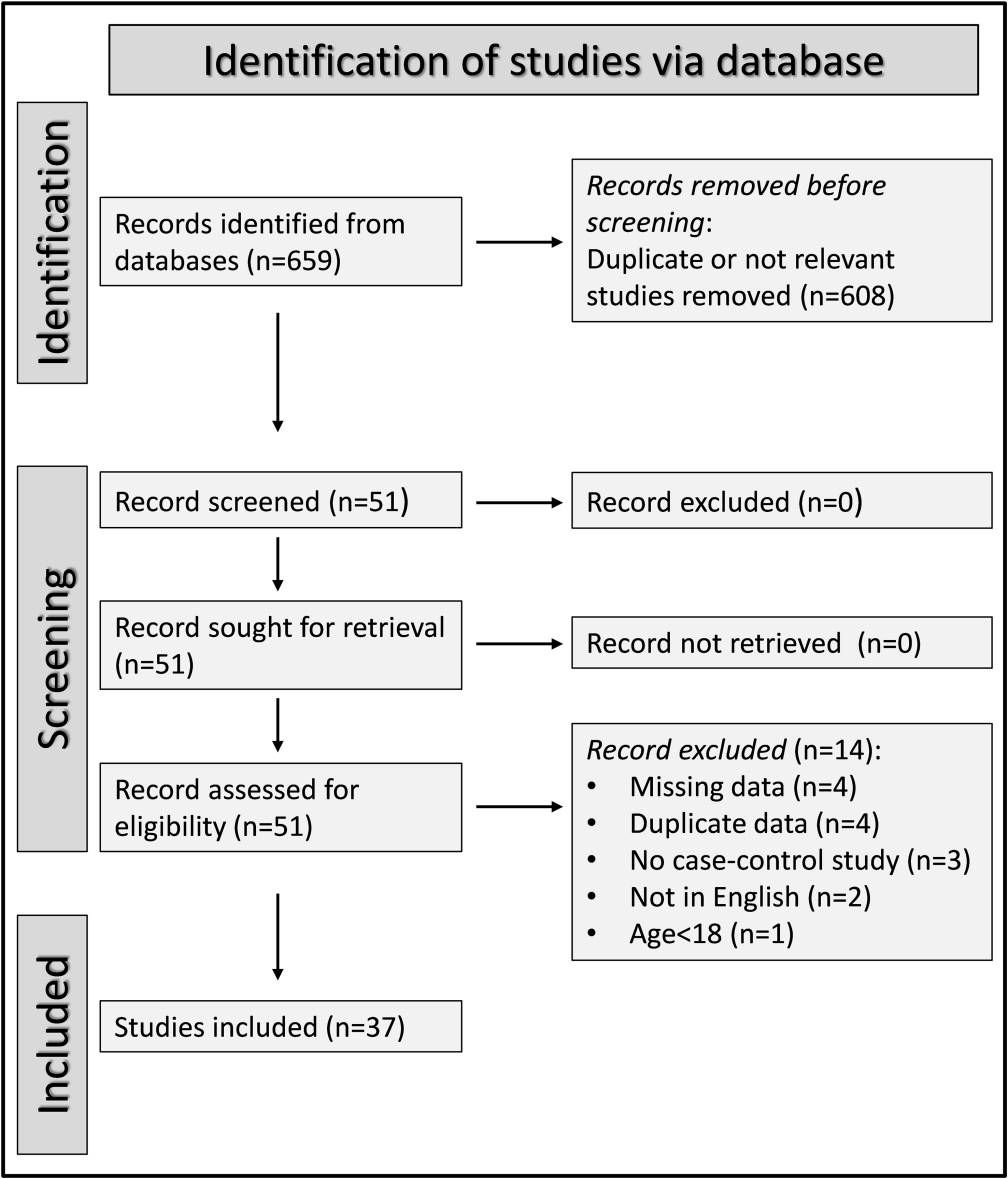
PRISMA 2020 flow diagram.

A flow chart describing the screening process is presented in **Figure 2**. We initially identified 659 articles. A total of 608 were excluded after the first screening because they were either duplicates or irrelevant. After a full-text review of the remaining 51 articles, a further 14 were excluded because of missing data (n=4), duplicate data (n=4), incorrect study design (n=3), non-English language used (n=2), and inclusion of children or adolescents (n=1). Therefore, 37 studies (43 study groups, 34 investigating plasma/serum, seven urine, one saliva, and one synovial fluid) were selected for analysis (**Table 1**) (54–90).

**Table 1:** Characteristics of the studies included in the meta-analysis.

				Healthy controls				Patients with RDs			
Study	Disease	Matrix	Method	n	Age (years)	M/F	Neopterin Mean ± SD (nmol/L)	n	Age (years)	M/F	Neopterin Mean ± SD (nmol/L)
Hannonen P et al., 1986, Finland (54)	RA	U	LC	67	NR	NR	218 ± 83^§^	67	53	14/53	342 ± 133^§^
Hagihara M et al. (a) 1990, Japan (55)	RA	S	LC	21	56	NR	26.13 ± 9.72	21	56	NR	21.63 ± 3.32
Hagihara M et al. (b) 1990, Japan (55)	SLE	S	LC	21	56	NR	26.13 ± 9.72	23	49	NR	43.08 ± 13.3
Krause A et al., 1990, Germany (56)	RA	SF	RIA	12	NR	NR	10.3 ± 25.0	17	48	6/11	41.0 ± 37.0
Leohirun L et al., 1991, Thailand (57)	SLE	U	LC	43	NR	NR	112 ± 40	43	18-42	7/36	925 ± 282
Lim KL et al., 1993, UK (58)	SLE	U	LC	65	45	2/63	158 ± 53	68	43	3/65	505 ± 326
Yoon J et al., 1993, Korea (59)	BD	S	RIA	30	NR	20/10	3.63 ± 0.88	67	38	34/33	6.36 ± 2.52
Altindag Z et al., 1994, Turkey (60)	BD	U	LC	14	20-34	7/7	125 ± 44	21	31	12/9	184 ± 119
Csipo I et al., 1995, Hungary (61)	SSc	S	ELISA	46	NR	NR	0.9 ± 2.3	29	50	NR	10.8 ± 4.5
Samsonov MY et al. (a) 1997, Austria (62)	DM	S	RIA	31	NR	NR	5.2 ± 1.8	15	35	NR	11.3 ± 4.6
Samsonov MY et al. (b) 1997, Austria (62)	PM	S	RIA	31	NR	NR	5.2 ± 1.8	13	39	NR	20.6 ± 11.3
Altindag ZZ et al., 1998, Turkey (63)	RA	U	LC	20	49	1/19	111 ± 34	36	50	2/34	331 ± 319
Andrys C et al., 1999, Czech Republic (64)	pSS	S	ELISA	26	NR	0/26	7.6 ± 2.3	17	58	2/15	17.9 ± 6.4
Keser G et al. (a) 2000, Turkey (65)	BD	S	ELISA	10	35	3	2.1 ± 0.7*	50	36	35/15	3.2 ± 1.9*
Keser G et al. (b) 2000, Turkey (65)	SLE	S	ELISA	10	35	NR	2.1 ± 0.7*	20	NR	NR	10.5 ± 8.5*
Kökçam I et al., 2002, Turkey (66)	BD	S	ELISA	25	NR	NR	12.16 ± 3.77*	25	31	13/12	17.34 ± 6.2*
Sfriso P et al. (a) 2003, Italy (67)	pSS	S	ELISA	20	48	0/20	5 ± 2.06	30	47	0/30	8.12 ± 3.36
Sfriso P et al. (b) 2003, Italy (67)	pSS	Sa	ELISA	20	48	0/20	2.83 ± 1.47	30	47	0/30	7.5 ± 7.61
de Castro MR et al., 2004, Brazil (68)	SLE	U	LC	49	NR	NR	295 ± 179	49	NR	NR	787 ± 145
Coskun B et al., 2005, Turkey (69)	BD	S	ELISA	30	32	15/15	8.7 ± 2.2*	40	33	21/19	14.3 ± 3.9*
Jin O et al., 2005, China (70)	SLE	S	ELISA	20	NR	NR	0.26 ± 0.19°	22	NR	NR	1.39 ± 1.1°
Mahmoud RAK et al., 2005, Egypt (71)	SLE	S	ELISA	10	26	0/10	5.76 ± 2.52	40	27	0/40	28.36 ± 13.19
Kose O et al., 2006, Turkey (72)	BD	S	LC	17	27	12/5	4.56 ± 0.45	68	26	64/4	7.74 ± 3.63
Ozkan S et al., 2007, Turkey (73)	BD	S	LC	21	39	6/15	12 ± 4.4	23	40	8/15	13.4 ± 3.6
Erturan I et al. (a) 2009, Turkey (74)	BD	S	ELISA	45	38	21/24	6.03 ± 3.46	45	39	21/24	12.68 ± 4.67
Erturan I et al. (b) 2009, Turkey (74)	BD	U	ELISA	45	38	21/24	104 ± 48	45	39	21/24	168 ± 149
Salem SAM et al., 2010, Egypt (75)	SLE	P	ELISA	20	26	2/18	9.4 ± 1.1	50	26	6/44	21.2 ± 5
Rho YH et al. (a) 2011, USA (76)	SLE	S	ELISA	177	47	45/232	5.87 ± 1.7	148	40	14/134	8.1 ± 2.44
Rho YH et al. (b) 2011, USA (76)	RA	S	ELISA	177	47	45/232	5.87 ± 1.7	166	54	52/114	6.97 ± 2.67
Bahrehmand F et al., 2012, Iran (77)	SLE	S	LC	101	37	22/82	6.5 ± 2.9	109	36	19/90	28.8 ± 38.1
Ozkan Y et al., 2012, Turkey (78)	RA	S	ELISA	20	62	4/16	7.14 ± 5.15	32	59	5/27	8.47 ± 7.8
D’Agostino LE et al., 2013, Italy (79)	RA	P	ELISA	38	37	9/29	5.62 ± 2.22	27	36	7/20	8.92 ± 4.83
Shahmohamadnejad S et al., 2015, Iran (80)	RA	P	LC	397	49	36/363	4.2 ± 2.22	419	50	42/377	5.93 ± 4.81
Gulkesen A et al., 2016, Turkey (81)	RA	S	ELISA	24	43	11/13	1.88 ± 1.84	33	53	9/24	23.98 ± 18.88
Baniamerian H et al., 2017, Iran (82)	SLE	S	LC	98	36	18/80	6.5 ± 2.9	100	37	20/80	25.7 ± 38.1
El-Lebedy D et al., 2017, Egypt (83)	RA	S	LC	100	NR	NR	4.74 ± 1.98	120	44	NR	11.46 ± 3.56
Tanhapour M et al., 2018, Iran (84)	SLE	S	LC	101	37	20/81	6.06 ± 2.08	107	36	19/88	12.77 ± 13.26
Zorbozan N et al., 2018, Turkey (85)	AS	S	ELISA	80	NR	NR	1.12 ± 0.32	160	NR	91/69	1.13 ± 0.39
Iranshahi N et al., 2019, Iran (86)	RA	P	ELISA	42	46	7/35	15.32 ± 9.02	47	51	7/40	17.63 ± 9.68
Akyurek F et al., 2020, Turkey (87)	BD	S	LC	54	37	NR	76.77 ± 38.27	57	36	NR	111.27 ± 37.49
Peng QL et al., 2020, China (88)	DM	S	ELISA	30	NR	NR	4.3 ± 2.0	182	NR	55/127	24.4 ± 15.8
Ekin S et al., 2021, Turkey (89)	RA	S	LC	30	50	11/19	4.19 ± 1.01*	30	52	10/20	25.99 ± 7.27*
Videm V et al., 2022, Norway (90)	RA	S	ELISA	3,415	58	2,053/1,362	5.15 ± 0.76	283	65	180/103	5.98 ± 0.88

AS, ankylosing spondylitis; BD, Behcet Disease; DM, dermatomyositis; ELISA, enzyme-linked immunosorbent assay; F, female, LC, liquid chromatography; M, male; NR, not reported; P, plasma; PM, polymyositis; pSS, primary Sjogren syndrome; RA, rheumatoid arthritis; RIA, radioimmunoassay; S, serum; Sa, saliva; SF, synovial fluid; SLE, systemic lupus erythematosus; SSc, systemic sclerosis; U, urine; ^§^, µmol/mol creatinine; *, ng/mL; °, µg/dL.

### Serum or plasma neopterin

#### Study characteristics

Thirty studies (34 study groups) reported serum or plasma neopterin concentrations in a total of 2,618 RD patients (mean age 43 years, 32% males) and 5,318 healthy controls (mean age 42 years, 47% males) (55, 59, 61, 64–67, 69–87, 89, 90).

Twenty studies were conducted in Asia (55, 59, 65, 66, 69, 70, 72–74, 77, 78, 80–82, 84–89), six in Europe (61, 62, 64, 67, 79, 90), three in Africa (71, 75, 83), and the remaining one in America (76). Ten study groups included patients with RA (55, 76, 78–81, 83, 86, 89, 90), nine with SLE (55, 65, 70, 71, 75–77, 82, 84), eight with BD (59, 65, 66, 69, 72–74, 87), three with IIM (62, 88), two with pSS (64, 67), one with SSc (61), and one with AS (85). The analytical methods used for measuring neopterin included an enzyme-linked immunosorbent assay (ELISA) in 18 studies (61, 64–67, 69–71, 74–76, 78, 79, 81, 85, 86, 88, 90), liquid chromatography with fluorimetric detection in 10 (55, 72, 73, 77, 80, 82–84, 87, 89), and radioimmunoassay in two (59, 62). Serum was analysed in 26 studies (55, 59, 61, 62, 64–67, 69–74, 76–78, 81–85, 87–90), and plasma in the remaining four (75, 79, 80, 86). RD duration, reported in 11 study groups, ranged between four and 11 years (61, 67, 71, 73–75, 78, 80, 81, 83, 90).

The risk of bias was low in 14 studies (61, 69, 75–77, 79, 81, 82, 84, 85, 87–90), and moderate in the remaining 16 (55, 59, 62, 64–67, 70–74, 78, 80, 83, 86) (**Supplementary Table 3**). All studies had an initially low certainty of evidence given the cross-sectional design (rating 2, ⊕⊕⊝⊝) (55, 59, 61, 64–67, 69–87, 89, 90).

#### Results of individual studies and syntheses

**Figure 3:**
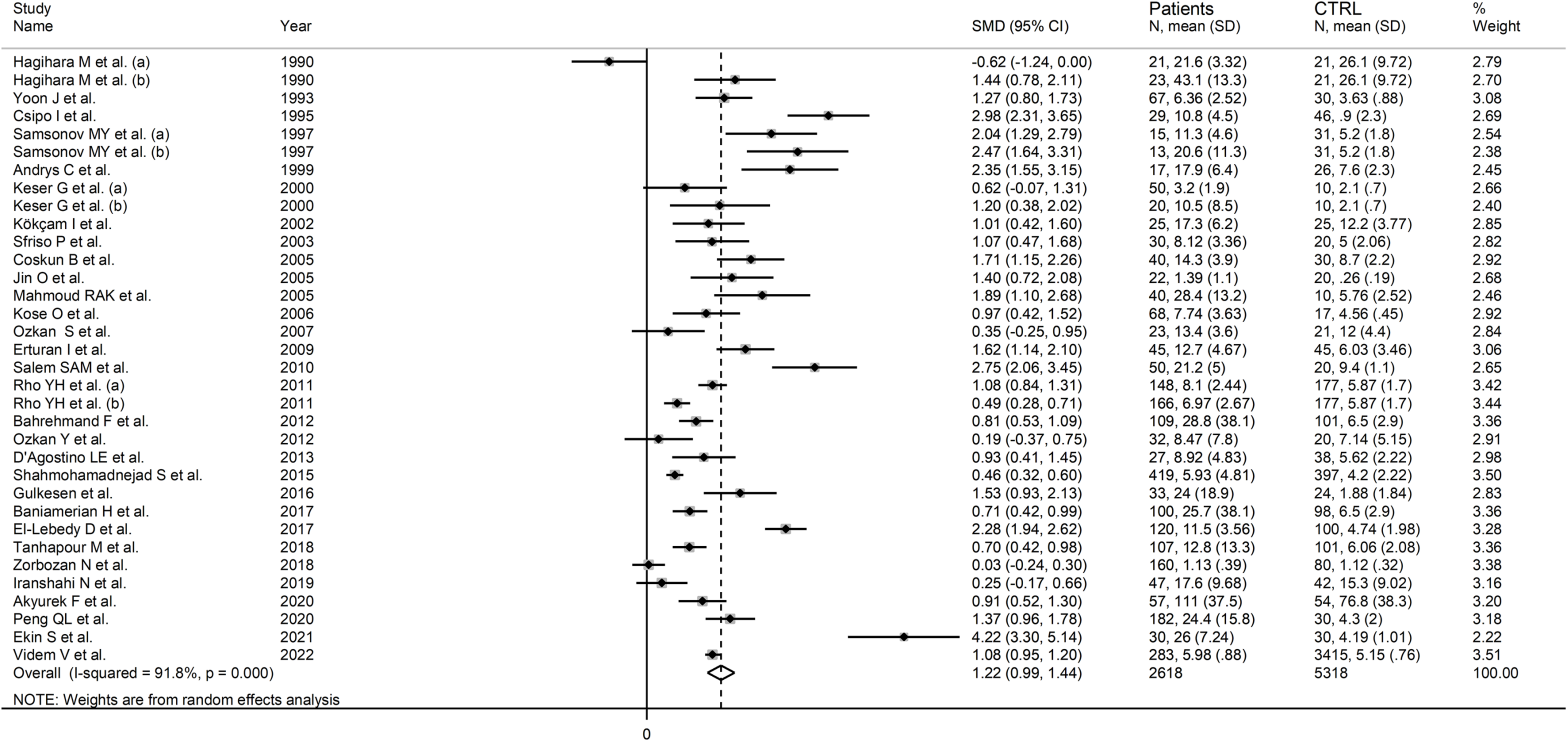
Forest plot of studies examining neopterin concentrations in RD patients and healthy controls in serum/plasma.

**Figure 4:**
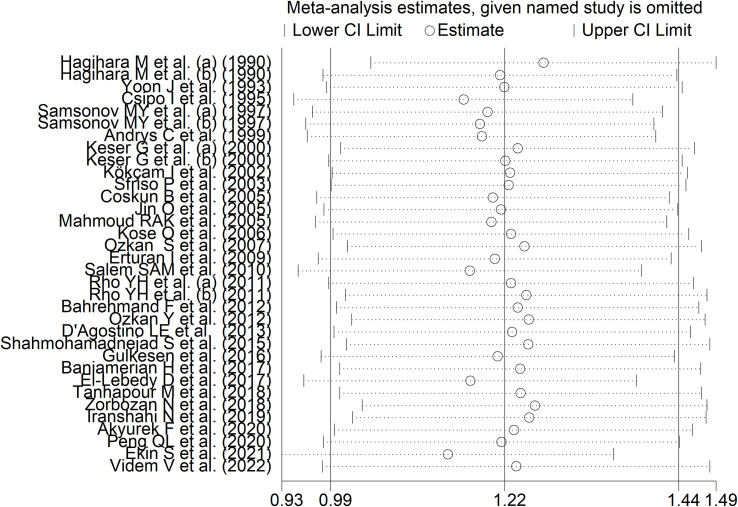
Sensitivity analysis of the association between neopterin and RDs in serum/plasma.

RD patients had significantly higher neopterin concentrations compared to healthy controls (SMD=1.22, 95% CI 0.99 to 1.44, p<0.001; I^2^ = 91.8%, p<0.001; **Figure 3**). In sensitivity analysis, the corresponding pooled SMD values were not influenced when individual studies were sequentially removed, with the effect size ranging between 1.14 and 1.27 (**Figure 4**). The effect size was also similar to the primary analysis after removing three studies accounting for 65% of the overall participant population (SMD=1.31, 95% CI 1.01 to 1.61; p<0.001; I^2^ = 91.2%, p<0.001) (76, 80, 90).

#### Publication bias

**Figure 5:**
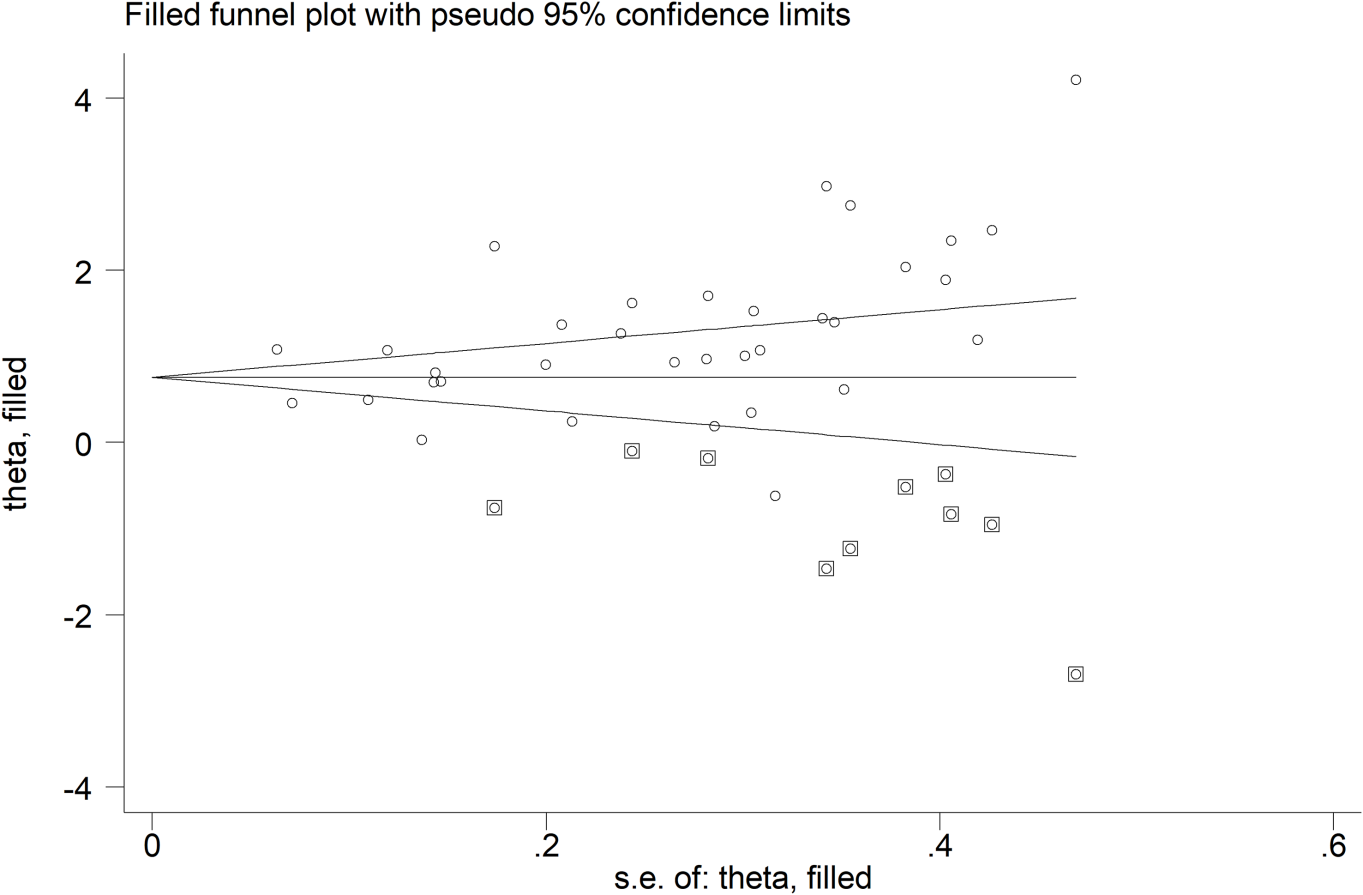
Funnel plot of studies investigating the association between neopterin and RDs in serum/plasma after “trimming-and-filling”. Enclosed circles and free circles indicate dummy studies and genuine studies, respectively.

A significant publication bias was observed (Begg’s test, p=0.004; Egger’s test, p=0.006). The “trim-and-fill” method identified ten missing studies to be added to the left side of funnel plot to ensure symmetry (**Figure 5**). The resulting effect side was attenuated yet still significant (SMD=0.78, 95% CI 0.54 to 1.02, p<0.001).

#### Meta-regression and sub-group and analysis

There were non-significant associations between the effect size and age (t=0.13, p=0.90), male to female ratio (t=-0.34, p=0.73), year of publication (t=-0.51, p=0.61), sample size (t=-0.53, p=0.60), disease duration (t=0.83, p=0.42), CRP (t=-0.50, p=0.62), or ESR (t=0.16, p=0.87).

**Figure 6:**
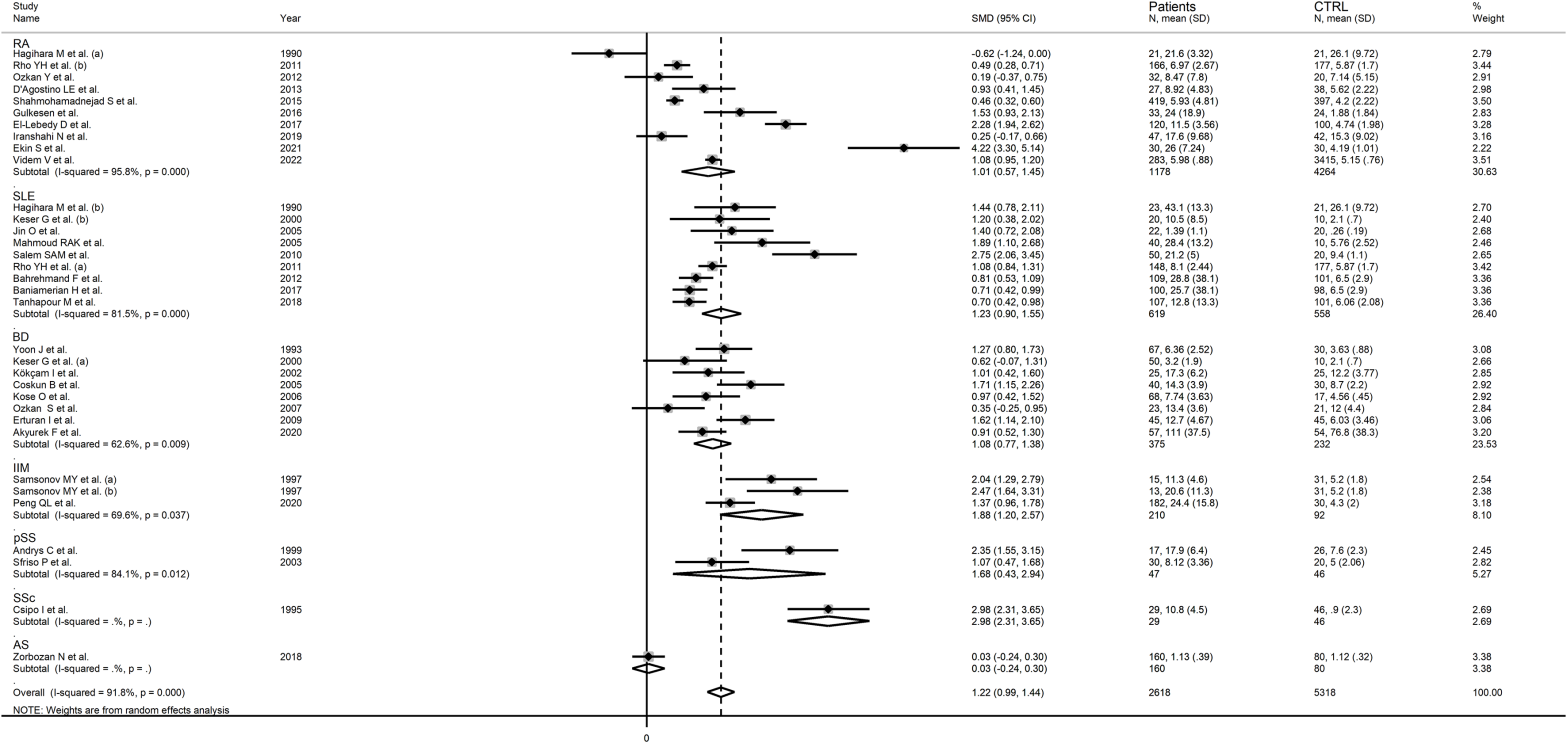
Forest plot of studies examining neopterin concentrations in RD patients and healthy controls in serum/plasma according to RD type.

**Figure 7:**
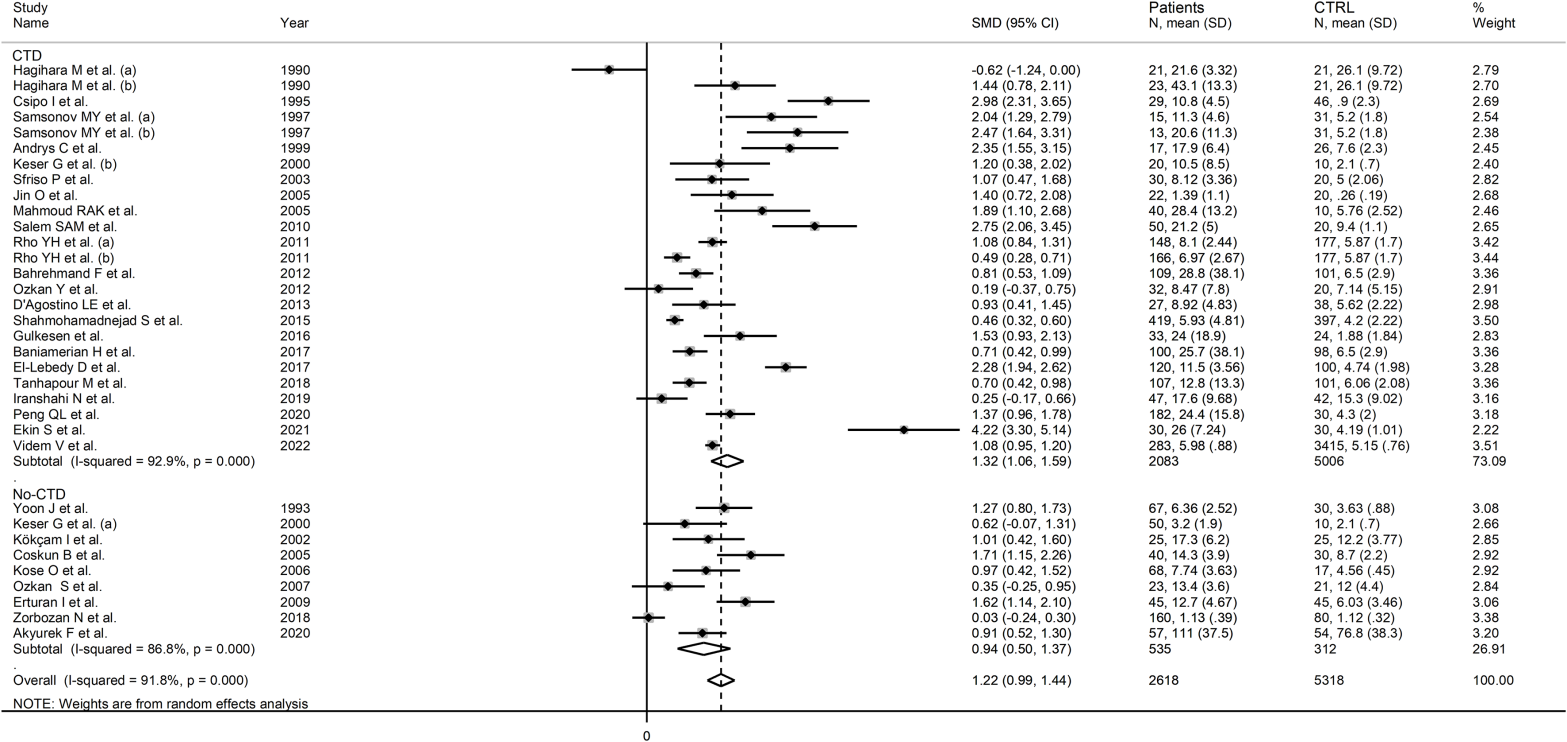
Forest plot of studies examining neopterin concentrations in RD patients and healthy controls in serum/plasma according to the presence of connective tissue disease.

**Figure 8:**
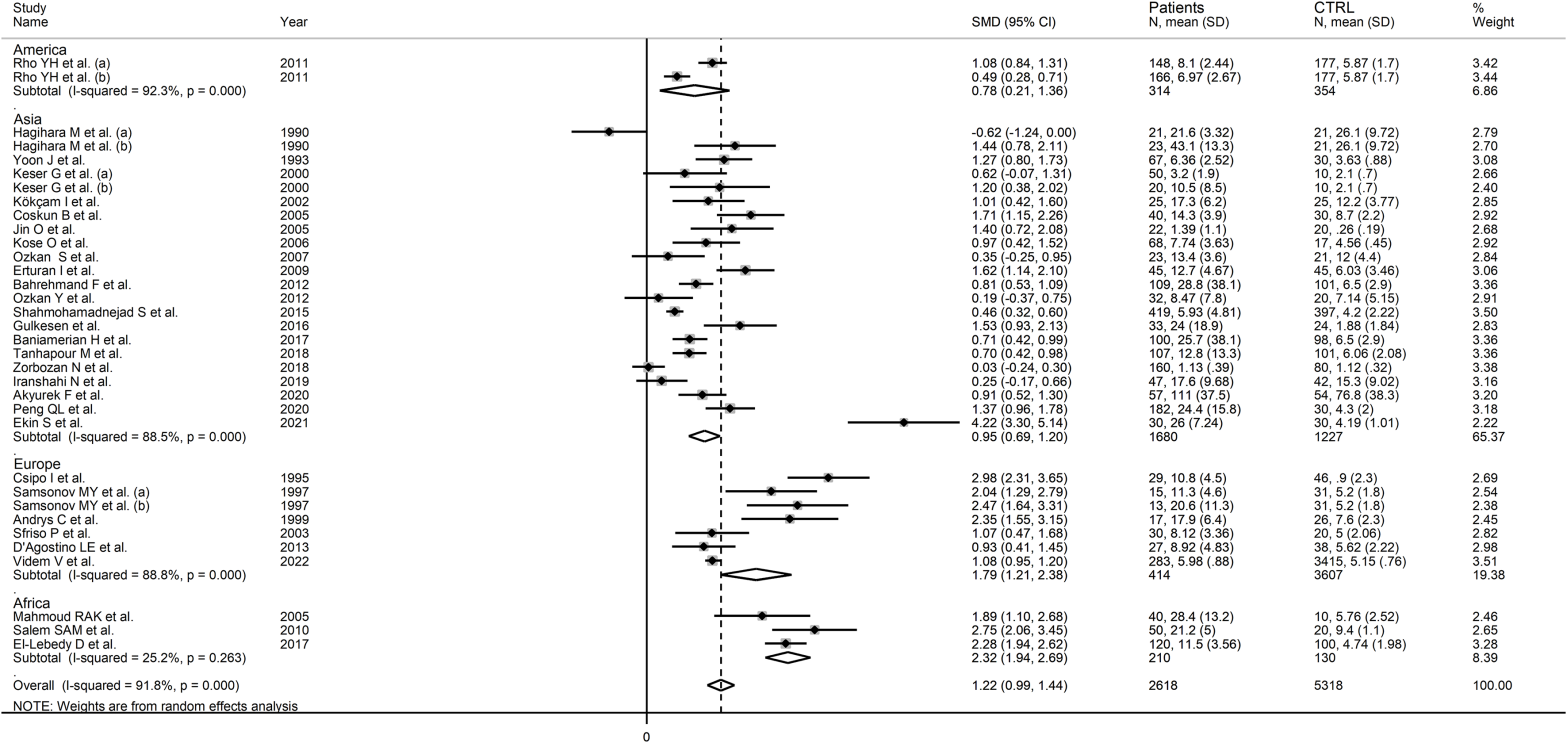
Forest plot of studies examining neopterin concentrations in RD patients and healthy controls in serum/plasma according to study continent.

**Figure 9:**
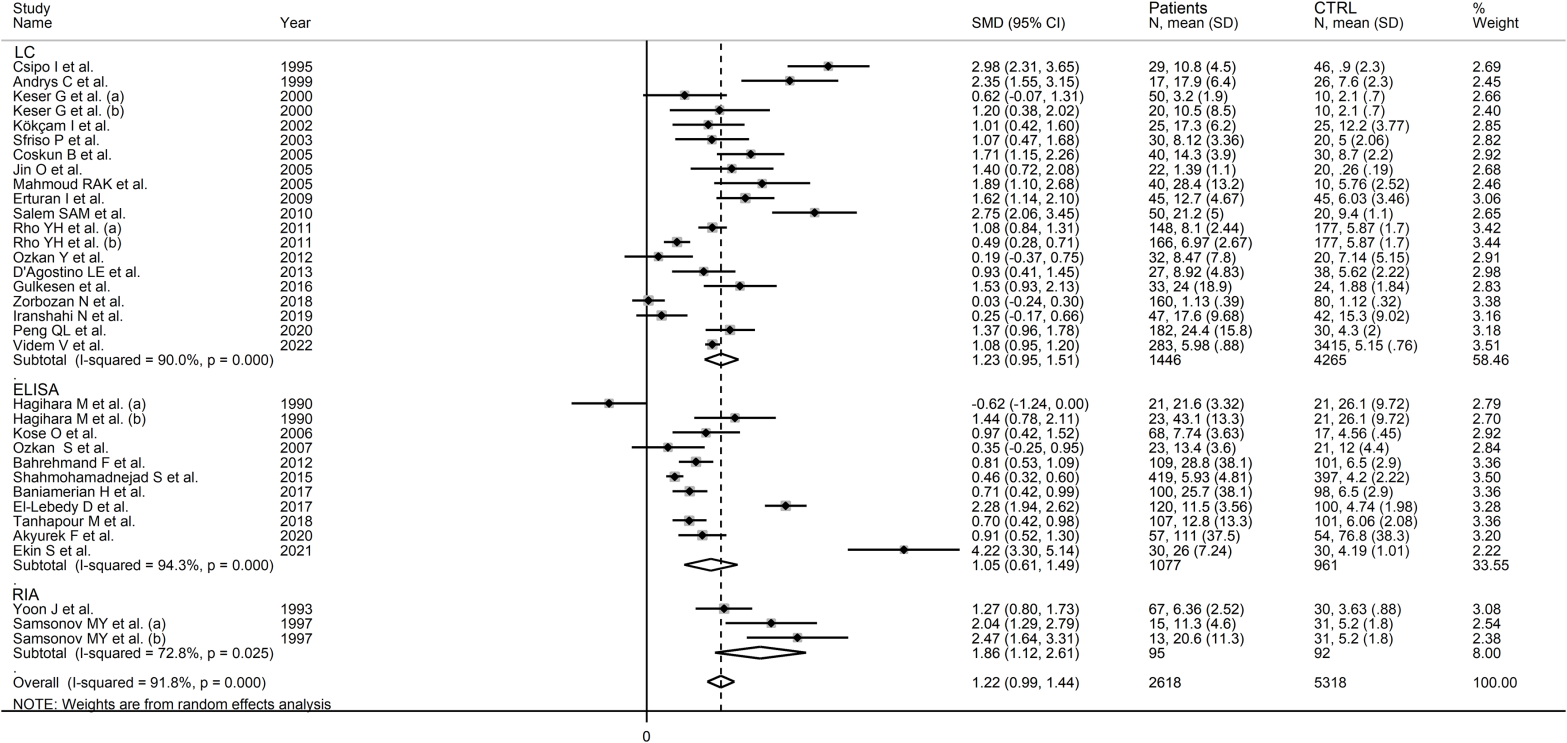
Forest plot of studies examining neopterin concentrations in RD patients and healthy controls in serum/plasma according to the analytical method used.

**Figure 10:**
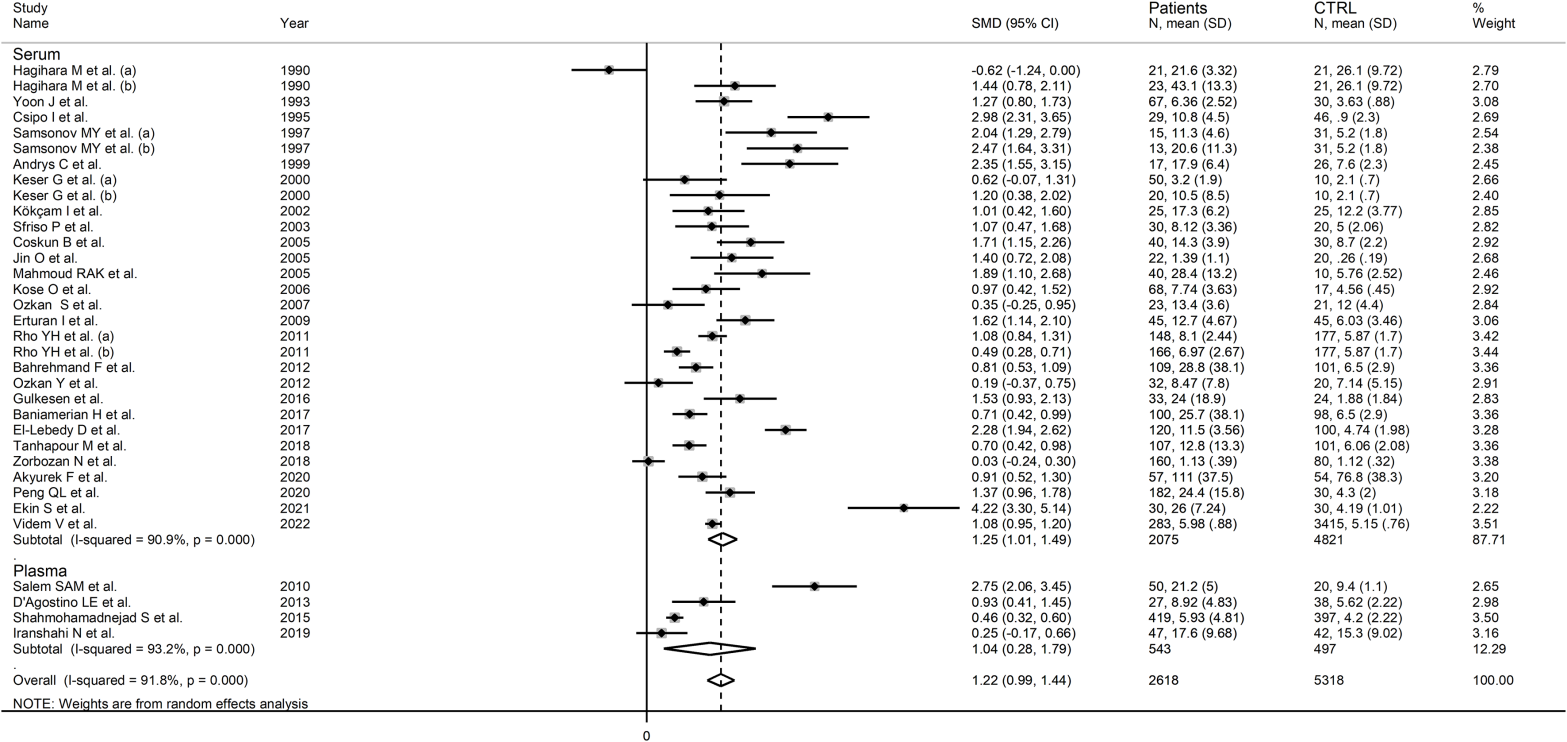
Forest plot of studies examining neopterin concentrations in RD patients and healthy controls in serum/plasma according to the sample matrix used for assessment (plasma or serum).

In subgroup analysis, there were non-significant differences (p=0.39) in SMD values between studies conducted in RA patients (SMD=1.01, 95% CI 0.57 to 1.45, p<0.001; I^2^ = 95.8%, p<0.001), SLE patients (SMD=1.23, 95% CI 0.90 to 1.55, p<0.001; I^2^ = 81.5%, p<0.001), BD patients (SMD=1.08, 95% CI 0.77 to 1.38, p<0.001; I^2^ = 62.6%, p=0.006), IIM patients (SMD=1.88, 95% CI 1.20 to 2.57, p<0.001; I^2^ = 69.6%, p=0.037), and pSS patients (SMD=1.68, 95% CI 0.43 to 2.94, p=0.008; I^2^ = 84.1%, p<0.001; **Figure 6**), with a lower heterogeneity observed in the BD and IIM subgroups. Similarly, the pooled SMD was non-significantly different (p=0.25) between studies conducted in patients with CTD (SMD=1.32, 95% CI 1.06 to 1.59, p<0.001; I^2^ = 92.9%, p<0.001) and without CTD (SMD=0.94, 95% CI 0.50 to 1.37, p<0.001; I^2^ = 92.0%, p<0.001; **Figure 7**). By contrast, a significant (p=0.003) increase in the effect size was observed between studies conducted in America (SMD=0.78, 95% CI 0.21 to 1.36, p=0.007; I^2^ = 92.3%, p<0.001), Asia (SMD=0.95, 95% CI 0.69 to 1.20, p<0.001; I^2^ = 88.5%, p<0.001), Europe (SMD=1.79, 95% CI 1.21 to 2.38, p<0.001; I^2^ = 88.8%, p<0.001) and Africa (SMD=2.32, 95% CI 1.94 to 2.69, p<0.001; I^2^ = 25.2%, p<0.263; **Figure 8**), with a lower heterogeneity observed in the African subgroup. There were non-significant differences (p=0.48) in pooled SMD between studies using liquid chromatography (SMD=1.23, 95% CI 0.95 to 1.51, p<0.001; I^2^ = 90.0%, p<0.001), ELISA (SMD=1.05, 95% CI 0.61 to 1.49, p<0.001; I^2^ = 94.3%, p<0.001), and RIA (SMD=1.86, 95% CI 1.12 to 2.61, p<0.001; I^2^ = 72.8%, p=0.025; **Figure 9**. Finally, non-significant differences (p=0.66) in pooled SMD were also observed between studies investigating serum (SMD=1.25, 95% CI 1.01 to 1.49, p<0.001; I^2^ = 90.9%, p<0.001) and plasma (SMD=1.04, 95% CI 0.28 to 1.79, p=0.007; I^2^ = 93.2%, p<0.001; **Figure 10**).

#### Certainty of evidence

The overall level of certainty was upgraded to moderate (rating 3, ⊕⊕⊕⊝) after taking into account the low-moderate risk of bias in all studies (no rating change), the high but partly explainable heterogeneity (no rating change), the lack of indirectness (no rating change), the relatively low imprecision (confidence intervals not crossing the threshold, no rating change), the large effect size (SMD=1.22, upgrade by one level), and the presence of publication bias which was corrected using the “trim-and-fill” method (no rating change).

### Urine neopterin

#### Study characteristics

Seven studies investigated urinary concentrations of neopterin in a total of 329 patients (mean age 46.4 years, 21.1% males) and 303 healthy controls (mean age 46.5 years, 20.5% males) (54, 57, 58, 60, 63, 68, 74). Four studies were conducted in Asia (57, 60, 63, 74), two in Europe (54, 58), and one in America (68). Three studies investigated patients with SLE (57, 58, 68), two with RA (54, 63), and two with BD (60, 74). Liquid chromatography with fluorimetric detection was used in six studies (54, 57, 58, 60, 63, 68), and ELISA in the remaining one (74).

The risk of bias was considered low in two studies (58, 63), moderate in two (57, 74), and high in the remaining three (54, 60, 68) (**Supplementary Table 3**). All studies had an initially low certainty of evidence given the cross-sectional design (rating 2, ⊕⊕⊝⊝) (54, 57, 58, 60, 63, 68, 74).

#### Results of individual studies and syntheses

**Figure 11:**
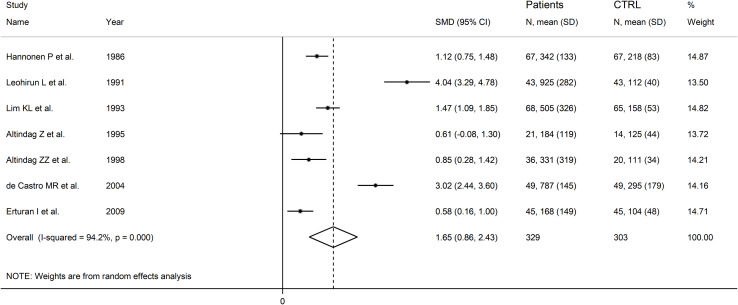
Forest plot of studies examining neopterin concentrations in RD patients and healthy controls in urine.

**Figure 12:**
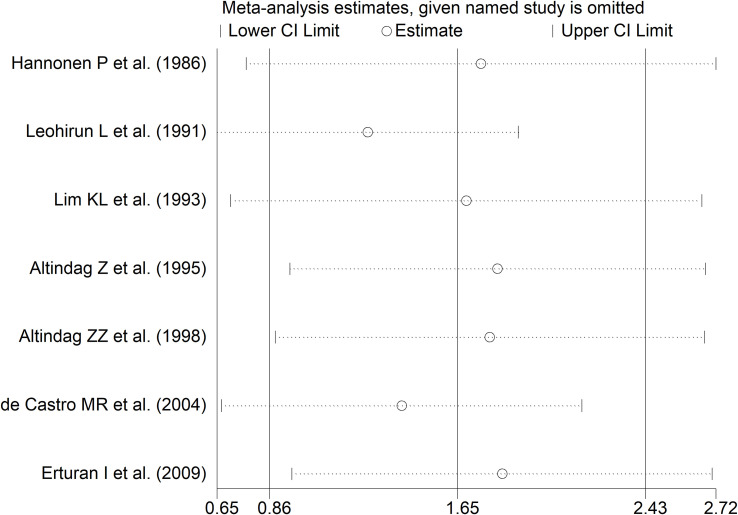
Sensitivity analysis of the association between neopterin and RDs in urine.

The forest plot showed that RD patients had significantly higher urinary neopterin concentrations compared to healthy controls (SMD=1.65, 95% CI 0.86 to 2.43, p<0.001; I^2^ = 94.2%, p<0.001; **Figure 11**). In sensitivity analysis, the corresponding pooled SMD values were not influenced when individual studies were sequentially removed, with the effect size ranging between 1.27 and 1.83 (**Figure 12**).

### Publication bias and meta-regression analysis

Assessment of publication bias and meta-regression could not be performed because of the small number of studies.

### Subgroup analysis

**Figure 13:**
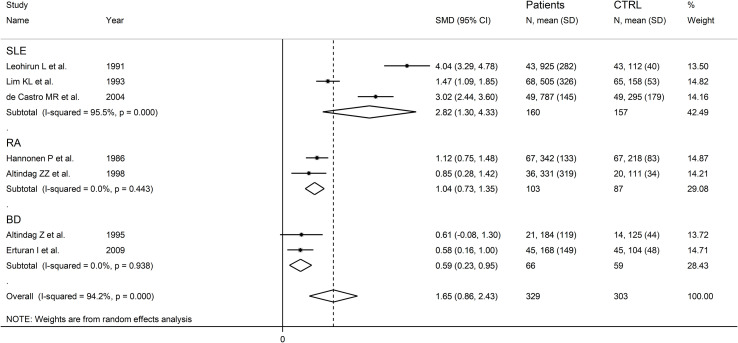
Forest plot of studies examining neopterin concentrations in RD patients and healthy controls in urine according to RD type.

**Figure 14:**
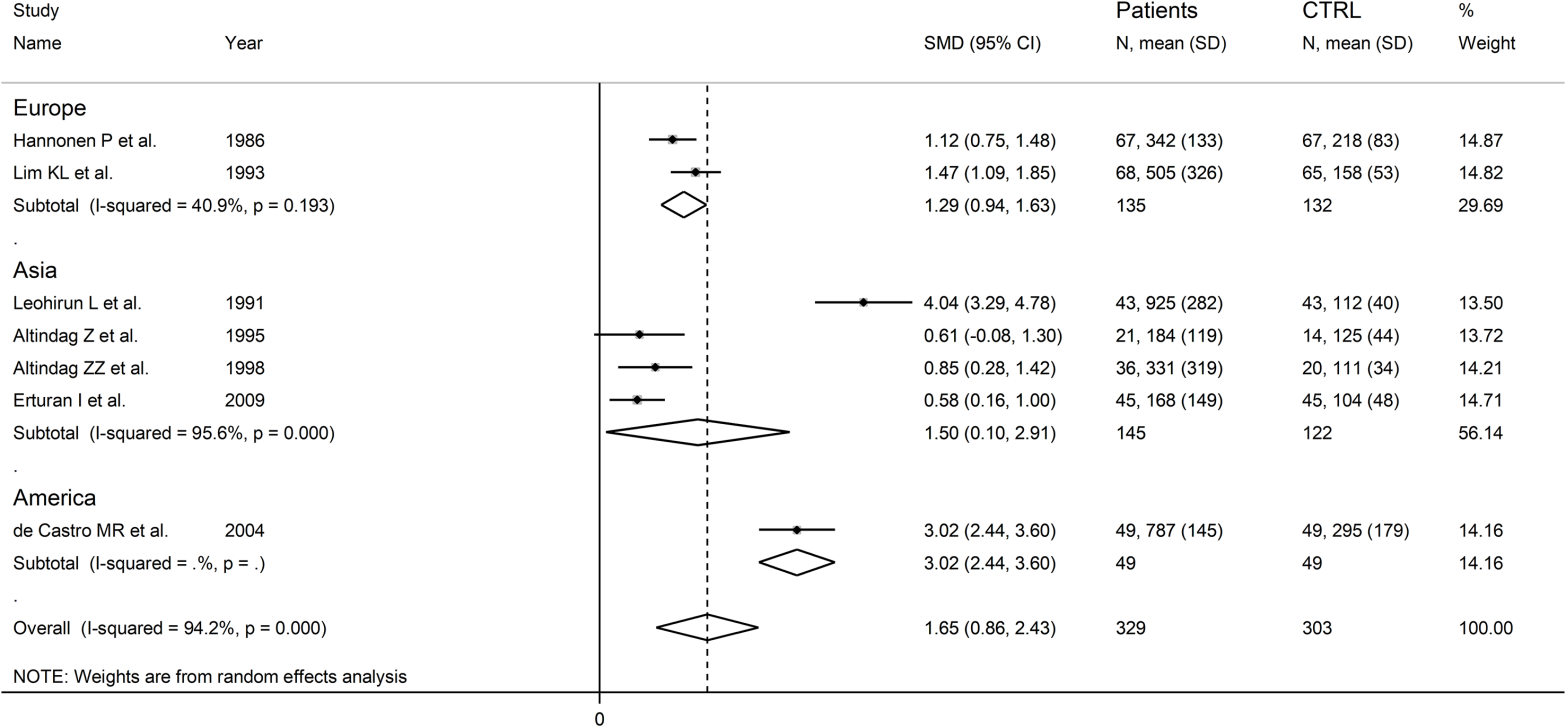
Forest plot of studies examining neopterin concentrations in RD patients and healthy controls in urine according to study continent.

There were significant differences (p=0.04) in SMD values between studies conducted in SLE patients (SMD=2.82, 95% CI 1.30 to 4.33, p<0.001; I^2^ = 95.5%, p<0.001), RA patients (SMD=1.04, 95% CI 0.73 to 1.35, p<0.001; I^2^ = 0.0%, p=0.44), and BD patients (SMD=0.59, 95% CI 0.23 to 0.95, p=0.001; I^2^ = 0.0%, p=0.94; **Figure 13**), with a virtual absence of heterogeneity in the RA and BD subgroups. By contrast, there were non-significant differences (p=0.40) in SMD values between European (SMD=1.29, 95% CI 0.94 to 1.63, p<0.001; I^2^ = 40.9%, p<0.001), and Asian studies (SMD=1.50, 95% CI 0.10 to 2.91, p<0.001; I^2^ = 94.2%, p<0.001; **Figure 14**), with a lower heterogeneity in the European subgroup.

#### Certainty of evidence

The overall level of certainty remained low (rating 2, ⊕⊕⊝⊝) after taking into account the low-moderate risk of bias in the majority of studies (no rating change), the high but partly explainable heterogeneity (no rating change), the lack of indirectness (no rating change), the relatively low imprecision (confidence intervals not crossing the threshold, no rating change), the large effect size (SMD=1.65, upgrade by one level), and lack of assessment of publication bias (downgrade one level).

#### Neopterin concentration in other biological fluids

One study reported significantly higher salivary concentrations of neopterin in pSS patients when compared with healthy subjects (9.5 ± 7.61 *vs*. 2.83 ± 1.47 nmol/L, p<0.005) (67), whereas another study reported that RA patients have increased concentrations of neopterin in synovial fluid when compared with healthy controls (41 ± 37 *vs*. 10.3 ± 25 nmol/L, p<0.001) (56) (**Table 1**).

## Discussion

The results of our systematic review and meta-analysis have shown that the plasma/serum and urinary concentrations of neopterin, a biomarker of interferon-γ activation, are significantly higher in patients with RDs compared to healthy controls. In meta-regression analysis, the effect size of the between-group differences in plasma/serum neopterin concentrations (SMD) was not associated with a range of study and patient characteristics, including age, male to female ratio, year of publication, study sample size, RD duration, CRP, and ESR. Similarly, in subgroup analysis the SMD was not associated with the type of RD (i.e., RA, SLE, BD, and pSS), the presence of CTD, the analytical method used to determine neopterin, or the matrix used for assessment (plasma *vs*. serum). By contrast, there was a significant association between the SMD (plasma or urine) and the study geographical location, with progressively higher SMD values in studies conducted in America, Asia, Europe, and Africa, and between the SMD (urine) and the type of RD investigated.

Taken together, these results suggest that neopterin can significantly discriminate between physiological states and different types of RD, including an autoimmune and/or an autoinflammatory component, using a range of analytical methods that can be applied both in plasma/serum and in urine. High-performance liquid chromatography with fluorimetric detection, ELISA, and RIA were the analytical methods most used to measure neopterin in biological fluids. High-performance liquid chromatography with fluorimetric detection offers a particularly high sensitivity, enabling the simultaneous detection of low neopterin concentrations. Its specificity is also high due to compound separation in the sample, which minimize the interference from other molecules. Quantitative accuracy is achievable, particularly when coupled with sensitive fluorimetric detection. However, it demands specialized equipment and expertise for operation and maintenance, and the process is time-consuming and potentially costly (91). ELISA is particularly suitable for the assessment of a large volume of samples due to its capacity to process multiple samples simultaneously. Its execution is relatively straightforward, with many commercially available kits. The broad dynamic range of quantitative values is an advantage, covering both low and high neopterin concentrations. However, specificity relies on the quality of antibodies used, and cross-reactivity with related compounds might limit accuracy. Additionally, sensitivity might be an issue with very low concentrations (38). RIA is known for its high sensitivity, enabling the detection of very low neopterin concentrations. Quantitative accuracy is attainable with proper optimization. Specificity depends on appropriately selected antibodies, which can be highly specific. However, there are also safety concerns due to the use of radioisotopes, requiring proper handling and disposal (92). RIA can involve complex steps due to the separation of bound and free fractions. Overall, the choice among these methods should be based on the required sensitivity, available resources, and safety considerations. High-performance liquid chromatography with fluorimetric detection offers high specificity and sensitivity but requires complex and costly equipment. ELISA is simple, high-capacity, and has a broad dynamic range, but specificity might be limited. RIA provides high sensitivity and precision but carries safety issues and has limitations in reagent availability.

Another interesting observation was the absence of significant correlations between the SMD of neopterin and CRP and ESR, biomarkers that are routinely used to assess inflammation and disease activity in RDs, also suggests that the information provided by neopterin can potentially complement existing knowledge to enhance diagnostic capacity. The presence of significant geographic-related and RD type-related differences in the SMD of neopterin also suggests the potential influence of ethnicity and specific RDs in mediating the associations between interferon-γ, macrophage activation, and inflammatory and immune pathways.

Although interferon-γ is mainly produced by T helper 1 and natural killer cells, macrophages can also contribute to its formation (93, 94). In this context, there is robust evidence that interferon-γ activates macrophages to the creation of a pro-inflammatory phenotype and, at the same time, stimulates the expression of pro-inflammatory cytokines and downregulates anti-inflammatory cytokines (**Figure 1**) (95, 96). Furthermore, interferon-γ regulates the initial steps of the adaptative immune response by influencing dendritic cells, T-cells, and B-cells (97–99). However, the excessive production of interferon-γ is responsible for the dysregulation of inflammatory and immune pathways, a phenomenon that has been observed in several hyperinflammatory disease states, cytokine release syndromes, and autoimmune conditions (28, 100–103). Notably, in these studies neopterin was measured as a biomarker of interferon-γ activity (28, 100–103). This pteridine analogue is not directly synthesized in macrophages, rather it is the oxidized form of another pteridine analogue synthesized in these cells, 7,8-dihydroneopterin. In activated macrophages, interferon-γ is responsible for the upregulation of GTP cyclohydrolase 1, the enzyme responsible for the bioconversion of GTP into 7,8-dihydroneopterin-triphosphate, which is then transformed to 7,8-dihydroneopterin by the action of phosphatase enzymes (**Figure 1**) (104–106). 7,8-dihydroneopterin is a known antioxidant and free radical scavenger with protective effects on low-density lipoprotein, other proteins, and lipids (107–109). The scavenging effects of 7,8-dihydroneopterin on free radicals lead to the synthesis of several oxidation products, including neopterin (**Figure 1**) (110, 111). Although 7,8-dihydroneopterin might theoretically serve as a robust biomarker of immune activation and redox state its physicochemical characteristics, particularly the low fluorescence, present analytical challenges when measured in isolation and in combination with neopterin (total neopterin) (40, 112, 113). Pending further analytical studies to optimize the measurement of 7,8-dihydroneopterin in blood and other biological samples, our systematic review and meta-analysis also warrants further studies to confirm the potential utility of neopterin specifically in the early detection of RDs. In this context, the absence of significant associations between the SMD of neopterin concentrations and RD duration observed in meta-regression analysis suggests that this biomarker can effectively discriminate between physiological states and presence of RDs also in patients with relatively short disease duration.

Another interesting observation was the presence of significant differences in the SMD of neopterin according to specific geographical locations. Epidemiological studies have shown that in healthy individuals neopterin concentrations can be influenced by age, body mass index, body composition and ethnicity (114, 115). In a study of 426 healthy subjects, black participants, particularly males, had significantly higher concentrations of neopterin than white participants (114). However, opposite results, with higher neopterin concentrations in white compared to black subjects, or no ethnic-related differences were reported in other studies (116, 117). A systematic review and meta-analysis has also investigated the association between a functional polymorphism of the *interferon-γ* gene, +874 T/A, associated with excess production of interferon-γ (118), and the risk of autoimmune disease. In this study, there were significant differences in the frequencies of the T allele across Asian (34.1%), Middle Eastern (47.8%), Latin American (51.5%), and Caucasian subjects (74.2%). Furthermore, the T allele was significantly associated with the risk of autoimmune disease in Latin Americans, but not in Middle Eastern, Asian, or Caucasian populations (119). Clearly, additional research is warranted to investigate the influence of ethnicity on interferon-γ production, macrophage activation, neopterin concentrations, and RDs. The additional observation that the SMD of urine neopterin was significantly associated with specific types of RD also requires further studies to investigate the capacity of urine neopterin to discriminate between different types of RD. At the same time, however, the significantly higher SMD of urine neopterin observed in studies of patients with SLE *vs*. other types of RD opens new opportunities to investigate the utility of this biomarker to diagnose and/or assess the severity of renal involvement, specifically nephritis, often observed in this group (120).

Our study has several strengths, including the assessment of neopterin in different biological fluids in a wide range of RD types, the study of associations between the effect size and several study and patient characteristics, and a rigorous evaluation of the risk of bias and the certainty of evidence. Significant limitations include the paucity of studies investigating specific types of RD (i.e., AS, SSc, FMF, and PsA), and the high heterogeneity observed. However, we identified potential sources of heterogeneity in subgroup analyses (type of RD and study continent for both plasma/serum and urine neopterin). Furthermore, sensitivity analysis ruled out the effect of individual studies on the overall effect size.

In conclusion, this systematic review and meta-analysis has shown the presence of significant alterations in the plasma/serum and urinary concentrations of neopterin, a biomarker of interferon-γ production, macrophage activation, inflammation, and oxidative stress, in patients with RD. Further research is warranted to determine the capacity of neopterin to identify early *vs*. overt RD manifestations and justify its introduction in clinical practice.

## Data availability statement

The raw data supporting the conclusions of this article will be made available by the authors, without undue reservation.

## Author contributions

AM: Conceptualization, Data curation, Methodology, Writing – original draft, Writing – review & editing. AZ: Conceptualization, Data curation, Formal Analysis, Investigation, Methodology, Validation, Writing – review & editing.
