# Supplementary material for: A systematic review and meta-analysis of neopterin in rheumatic diseases
Source: Front Immunol. 2023 Sep 18;14:1271383. doi: 10.3389/fimmu.2023.1271383 (PMC10548830; doi:10.3389/fimmu.2023.1271383)
Supplement: Supplementary file 3 [file Table_3.docx]

**Supplementary Table 3.** The Joanna Briggs Institute critical appraisal checklist.

| **Study** | **Were the criteria for inclusion clearly defined?** | **Were the subjects and the setting described in detail?** | **Was the exposure measured in a valid and reliable way?** | **Were objective, standard criteria used for measurement of the condition?** | **Were confounding factors identified?** | **Were strategies to deal with confounding factors stated?** | **Were the outcomes measured in a valid and reliable way?** | **Was appropriate statistical analysis used?** | **Risk of bias** |
| --- | --- | --- | --- | --- | --- | --- | --- | --- | --- |
| Hannonen P (1) | No | No | Yes | No | No | No | Yes | Yes | High |
| Hagihara M (2) | No | Yes | Yes | Yes | No | No | Yes | Yes | Moderate |
| Krause A (3) | Yes | Yes | Yes | Yes | Yes | Yes | Yes | Yes | Low |
| Leohirun L (4) | No | Yes | Yes | Yes | No | No | Yes | Yes | Moderate |
| Lim KL (5) | Yes | Yes | Yes | Yes | Yes | Yes | Yes | Yes | Low |
| Yoon J (6) | No | Yes | Yes | Yes | No | No | Yes | Yes | Moderate |
| Altindag Z (7) | No | No | Yes | No | No | No | Yes | Yes | High |
| Csipo I (8) | Yes | Yes | Yes | Yes | No | No | Yes | Yes | Low |
| Samsonov MY (9) | No | Yes | Yes | Yes | No | No | Yes | Yes | Moderate |
| Altindag ZZ (10) | Yes | Yes | Yes | Yes | No | No | Yes | Yes | Low |
| Andrys C (11) | No | Yes | Yes | Yes | No | No | Yes | Yes | Moderate |
| Keser G (12) | No | Yes | Yes | Yes | No | No | Yes | Yes | Moderate |
| Kökçam I (13) | No | Yes | Yes | Yes | No | No | Yes | Yes | Moderate |
| Sfriso P (14) | No | Yes | Yes | Yes | No | No | Yes | Yes | Moderate |
| de Castro MR (15) | No | No | No | No | No | No | Yes | Yes | High |
| Coskun B (16) | Yes | Yes | Yes | Yes | No | No | Yes | Yes | Low |
| Jin O (17) | No | Yes | Yes | Yes | No | No | Yes | Yes | Moderate |
| Mahmoud RAK (18) | No | Yes | Yes | Yes | No | No | Yes | Yes | Moderate |
| Kose O (19) | No | Yes | Yes | Yes | No | No | Yes | Yes | Moderate |
| Ozkan S (20) | No | Yes | Yes | Yes | No | No | Yes | Yes | Moderate |
| Erturan I (21) | No | Yes | Yes | Yes | No | No | Yes | Yes | Moderate |
| Salem SAM (22) | Yes | Yes | Yes | Yes | No | No | Yes | Yes | Low |
| Rho YH (23) | Yes | Yes | Yes | Yes | Yes | Yes | Yes | Yes | Low |
| Bahrehmand F (24) | Yes | Yes | Yes | Yes | No | No | Yes | Yes | Low |
| Ozkan Y (25) | No | Yes | Yes | Yes | No | No | Yes | Yes | Moderate |
| D'Agostino LE (26) | Yes | Yes | Yes | Yes | No | No | Yes | Yes | Low |
| Shahmohamadnejad S (27) | No | Yes | Yes | Yes | No | No | Yes | Yes | Moderate |
| Gulkesen A (28) | Yes | Yes | Yes | Yes | No | No | Yes | Yes | Low |
| Baniamerian H (29) | Yes | Yes | Yes | Yes | No | No | Yes | Yes | Low |
| El-Lebedy D (30) | No | Yes | Yes | Yes | No | No | Yes | Yes | Moderate |
| Tanhapour M (31) | Yes | Yes | Yes | Yes | No | No | Yes | Yes | Low |
| Zorbozan N (32) | Yes | Yes | Yes | Yes | No | No | Yes | Yes | Low |
| Iranshahi N (33) | No | Yes | Yes | Yes | No | No | Yes | Yes | Moderate |
| Akyurek F (34) | Yes | Yes | Yes | Yes | No | No | Yes | Yes | Low |
| Peng QL (35) | Yes | Yes | Yes | Yes | Yes | Yes | Yes | Yes | Low |
| Ekin S (36) | Yes | Yes | Yes | Yes | No | No | Yes | Yes | Low |
| Videm V (37) | No | Yes | Yes | Yes | Yes | Yes | Yes | Yes | Low |

**References**

1. Hannonen P, Tikanoja S, Hakola M, Mottonen T, Viinikka L, Oka M. Urinary neopterin index as a measure of rheumatoid activity. Scand J Rheumatol. 1986;15(2):148-52. doi: 10.3109/03009748609102081

2. Hagihara M, Nagatsu T, Ohhashi M, Miura T. Concentrations of neopterin and biopterin in serum from patients with rheumatoid arthritis or systemic lupus erythematosus and in synovial fluid from patients with rheumatoid or osteoarthritis. Clin Chem. 1990;36(4):705-6. doi:

3. Krause A, Protz H, Goebel KM. Correlation between synovial neopterin and inflammatory activity in rheumatoid arthritis. Ann Rheum Dis. 1989;48(8):636-40. doi: 10.1136/ard.48.8.636

4. Leohirun L, Thuvasethakul P, Sumethkul V, Pholcharoen T, Boonpucknavig V. Urinary neopterin in patients with systemic lupus erythematosus. Clin Chem. 1991;37(1):47-50. doi:

5. Lim KL, Jones AC, Brown NS, Powell RJ. Urine neopterin as a parameter of disease activity in patients with systemic lupus erythematosus: comparisons with serum sIL-2R and antibodies to dsDNA, erythrocyte sedimentation rate, and plasma C3, C4, and C3 degradation products. Ann Rheum Dis. 1993;52(6):429-35. doi: 10.1136/ard.52.6.429

6. Yoon J, Lee SH, Bang D, Lee S, Kim JC, Chung TH. Elevated Serum Levels of Neopterin in Patients with Behçet's Disease. Ann Dermatol. 1993;5(2):74-8. doi: <https://doi.org/10.5021/ad.1993.5.2.74>

7. Altindağ Z, Şahin G, Akpek G, Koç Y, Işimer A, Kansu E, et al. Urinary Neopterin Levels as an Indicator of Disease Activation in Behçet’s Disease. 1995;6:79-83. doi:

8. Csipo I, Czirjak L, Szanto S, Szerafin L, Sipka S, Szegedi G. Decreased serum tryptophan and elevated neopterin levels in systemic sclerosis. Clin Exp Rheumatol. 1995;13(2):269-70. doi:

9. Samsonov MY, Nassonov EL, Tilz GP, Geht BM, Demel U, Gurkina GT, et al. Elevated serum levels of neopterin in adult patients with polymyositis/dermatomyositis. Br J Rheumatol. 1997;36(6):656-60. doi: 10.1093/rheumatology/36.6.656

10. Altindag ZZ, Sahin G, Inanici F, Hascelik Z. Urinary neopterin excretion and dihydropteridine reductase activity in rheumatoid arthritis. Rheumatol Int. 1998;18(3):107-11. doi: 10.1007/s002960050067

11. Andrys C, Krejsek J, Slezak R, Drahosova M, Kopecky O. Serum soluble adhesion molecules (sICAM-1, sVCAM-1, sE-selectin) and neopterin in patients with Sjogren's syndrome. Acta Medica (Hradec Kralove). 1999;42(3):97-101. doi:

12. Keser G, Oksel F, Aksu K, Kabasakal Y, Gumusdis G, Doganavs argil E, et al. Serum neopterin levels in Behcet's syndrome. Clin Rheumatol. 2000;19(4):328-9. doi:

13. Kokcam I, Naziroglu M. Effects of vitamin E supplementation on blood antioxidants levels in patients with Behcet's disease. Clin Biochem. 2002;35(8):633-9. doi: 10.1016/s0009-9120(02)00400-9

14. Sfriso P, Ostuni P, Botsios C, Andretta M, Oliviero F, Punzi L, et al. Serum and salivary neopterin and interferon-gamma in primary Sjogren's syndrome. Correlation with clinical, laboratory and histopathologic features. Scand J Rheumatol. 2003;32(2):74-8. doi: 10.1080/03009740310000067

15. de Castro MR, Di Marco GS, Arita DY, Teixeira LC, Pereira AB, Casarini DE. Urinary neopterin quantification by reverse-phase high-performance liquid chromatography with ultraviolet detection. J Biochem Biophys Methods. 2004;59(3):275-83. doi: 10.1016/j.jbbm.2004.03.004

16. Coskun B, Saral Y, Godekmerdan A, Erden I, Coskun N. Activation markers in Behcet's disease. Skinmed. 2005;4(5):282-6. doi: 10.1111/j.1540-9740.2005.03865.x

17. Jin O, Sun LY, Zhou KX, Zhang XS, Feng XB, Mok MY, et al. Lymphocyte apoptosis and macrophage function: correlation with disease activity in systemic lupus erythematosus. Clin Rheumatol. 2005;24(2):107-10. doi: 10.1007/s10067-004-0972-x

18. Mahmoud RA, El-Gendi HI, Ahmed HH. Serum neopterin, tumor necrosis factor-alpha and soluble tumor necrosis factor receptor II (p75) levels and disease activity in Egyptian female patients with systemic lupus erythematosus. Clin Biochem. 2005;38(2):134-41. doi: 10.1016/j.clinbiochem.2004.11.002

19. Kose O, Arca E, Akgul O, Erbil K. The levels of serum neopterin in Behcet's disease--objective marker of disease activity. J Dermatol Sci. 2006;42(2):128-30. doi: 10.1016/j.jdermsci.2006.02.001

20. Ozkan Y, Yardim-Akaydin S, Sepici A, Engin B, Sepici V, Simsek B. Assessment of homocysteine, neopterin and nitric oxide levels in Behcet's disease. Clin Chem Lab Med. 2007;45(1):73-7. doi: 10.1515/CCLM.2007.018

21. Erturan I, Basak PY, Ozturk O, Ceyhan AM, Akkaya VB. Is there any relationship between serum and urine neopterin and serum interferon-gamma levels in the activity of Behcet's disease? J Eur Acad Dermatol Venereol. 2009;23(12):1414-8. doi: 10.1111/j.1468-3083.2009.03334.x

22. Salem SA, Farouk HM, Mostafa AA, Hassan IM, Osman WM, Al-Shamy HA, et al. Keratinocyte and lymphocyte apoptosis: relation to disease outcome in systemic lupus erythematosus patients with and without cutaneous manifestations. Eur J Dermatol. 2010;20(1):35-41. doi: 10.1684/ejd.2010.0812

23. Rho YH, Solus J, Raggi P, Oeser A, Gebretsadik T, Shintani A, et al. Macrophage activation and coronary atherosclerosis in systemic lupus erythematosus and rheumatoid arthritis. Arthritis Care Res (Hoboken). 2011;63(4):535-41. doi: 10.1002/acr.20365

24. Bahrehmand F, Vaisi-Raygani A, Kiani A, Rahimi Z, Tavilani H, Navabi SJ, et al. Matrix metalloproteinase-2 functional promoter polymorphism G1575A is associated with elevated circulatory MMP-2 levels and increased risk of cardiovascular disease in systemic lupus erythematosus patients. Lupus. 2012;21(6):616-24. doi: 10.1177/0961203312436857

25. Ozkan Y, Mete G, Sepici-Dincel A, Sepici V, Simsek B. Tryptophan degradation and neopterin levels in treated rheumatoid arthritis patients. Clin Rheumatol. 2012;31(1):29-34. doi: 10.1007/s10067-011-1767-5

26. D'Agostino L E, Ventimiglia F, Verna JA, Colina Ade L, Aguirre Y, Arturi A, et al. Correlation between DAS-28 and neopterin as a biochemical marker of immune system activation in early rheumatoid arthritis. Autoimmunity. 2013;46(1):44-9. doi: 10.3109/08916934.2012.722143

27. Shahmohamadnejad S, Vaisi-Raygani A, Shakiba Y, Kiani A, Rahimi Z, Bahrehmand F, et al. Association between butyrylcholinesterase activity and phenotypes, paraoxonase192 rs662 gene polymorphism and their enzymatic activity with severity of rheumatoid arthritis: correlation with systemic inflammatory markers and oxidative stress, preliminary report. Clin Biochem. 2015;48(1-2):63-9. doi: 10.1016/j.clinbiochem.2014.08.016

28. Gulkesen A, Akgol G, Tuncer T, Kal GA, Telo S, Poyraz AK, et al. Relationship Between Leptin and Neopterin Levels and Disease Activation Parameters in Patients With Rheumatoid Arthritis. Arch Rheumatol. 2016;31(4):333-9. doi: 10.5606/ArchRheumatol.2016.5893

29. Baniamerian H, Bahrehmand F, Vaisi-Raygani A, Rahimi Z, Pourmotabbed T. Angiotensin type 1 receptor A1166C polymorphism and systemic lupus erythematosus: correlation with cellular immunity and oxidative stress markers. Lupus. 2017;26(14):1534-9. doi: 10.1177/0961203317711008

30. El-Lebedy D, Hussein J, Ashmawy I, Mohammed AM. Serum level of neopterin is not a marker of disease activity in treated rheumatoid arthritis patients. Clin Rheumatol. 2017;36(9):1975-9. doi: 10.1007/s10067-016-3433-4

31. Tanhapour M, Miri A, Vaisi-Raygani A, Bahrehmand F, Kiani A, Rahimi Z, et al. Synergism between apolipoprotein E E4 allele and paraoxonase (PON1) 55-M allele is associated with risk of systemic lupus erythematosus. Clin Rheumatol. 2018;37(4):971-7. doi: 10.1007/s10067-017-3859-3

32. Zorbozan N, Demir S, Çobankara V. Evaluation of the relationship between TNFα, sTNFR1, sTNFR2, sIL2R, IL6, neopterin with disease activity in ankylosing spondylitis. Turkish Journal of Biochemistry. 2018;43(5):487-94. doi: 10.1515/tjb-2017-0350

33. Iranshahi N, Assar S, Amiri SM, Zafari P, Fekri A, Taghadosi M. Decreased Gene Expression of Epstein-Barr Virus-Induced Gene 3 (EBI-3) may Contribute to the Pathogenesis of Rheumatoid Arthritis. Immunol Invest. 2019;48(4):367-77. doi: 10.1080/08820139.2018.1549066

34. Akyurek F, Tuncez Akyurek F. Investigation of pregnancy associated plasma protein-A and neopterin levels in Behcet's patients. Dermatol Ther. 2020;33(4):e13443. doi: 10.1111/dth.13443

35. Peng QL, Zhang YM, Liang L, Liu X, Ye LF, Yang HB, et al. A high level of serum neopterin is associated with rapidly progressive interstitial lung disease and reduced survival in dermatomyositis. Clin Exp Immunol. 2020;199(3):314-25. doi: 10.1111/cei.13404

36. Ekin S, Sivrikaya A, Akdag T, Yilmaz S, Gulcemal S. Elevated levels of neopterin and pentraxin 3 in patients with rheumatoid arthritis. Horm Mol Biol Clin Investig. 2021;42(4):419-23. doi: 10.1515/hmbci-2021-0012

37. Videm V, Houge IS, Liff MH, Hoff M. Inflammation mediates approximately one quarter of excess relative all-cause mortality in persons with rheumatoid arthritis: the Trondelag Health Study. Sci Rep. 2022;12(1):18599. doi: 10.1038/s41598-022-21977-9
